# Marine ecosystem connectivity mediated by migrant–resident interactions and the concomitant cross‐system flux of lipids

**DOI:** 10.1002/ece3.2167

**Published:** 2016-05-19

**Authors:** Mikael van Deurs, Anders Persson, Martin Lindegren, Charlotte Jacobsen, Stefan Neuenfeldt, Christian Jørgensen, P. Anders Nilsson

**Affiliations:** ^1^ Department of Biology ‐ Aquatic EcologyLund UniversityEcology BuildingSE‐223 62LundSweden; ^2^ National Institute for Aquatic resources ‐ Section for Marine Living ResourcesTechnical University of DenmarkJægersborgs alle 1DK‐2920CharlottenlundDenmark; ^3^ National Food InstituteTechnical University of DenmarkSøltofts pladsBuilding 221DK‐2800 Kgs.LyngbyDenmark; ^4^ Department of Environmental and Life Sciences ‐ BiologyKarlstad UniversitySE‐651 88KarlstadSweden; ^5^ Department of Biology ‐ Theoretical Ecology Group and Hjort Centre for Marine Ecosystem DynamicsUniversity of BergenN‐5020BergenNorway

**Keywords:** Arachidonic acid, Baltic Sea, *Clupea harengus*, dietary fatty acids, FATM, food quality, *Gadus morhua*, meta‐ecosystem, migration, nutritional quality, predator–prey interactions, resource subsidies, trophic tracers

## Abstract

Accumulating research argues that migrants influence the functioning and productivity of local habitats and ecosystems along migration routes and potentially drive cross‐system energy fluxes of considerable magnitude, yet empirical documentation of local ecological effects and descriptions of the underlying mechanisms are surprisingly rare. In this study, we discovered migrant–resident interactions and substantial cross‐system lipid transportation in the transition zone between the Baltic Sea and the North Sea where a resident cod population (predators) was found to interact with a herring population (prey) on a seasonal basis. We traced the lipids, using fatty acid trophic markers (FATM), from the herring feeding grounds in the North Sea to the cod livers in the Western Baltic Sea. Time series analysis of population dynamics indicated that population‐level production of cod is positively affected by the herring subsidies. However, the underlying mechanisms were more complicated than anticipated. During the herring season, large cod received most of its dietary lipids from the herring, whereas smaller cod were prevented from accessing the lipid pool due to a mismatch in predator–prey size ratio. Furthermore, while the herring were extremely rich in bulk energy, they were surprisingly poor in a specific functional fatty acid. Hence, our study was the first to illustrate how the magnitude cross‐system fluxes of subsidies in migrant–resident systems are potentially constrained by the size structure of the resident predator population and the nutritional quality of the migrants.

## Introduction

It is reasonable to hypothesize that migrants are strongly implicated in the functioning and productivity of local habitats or ecosystems positioned along their migration routes, where migrants exert trophic influence or carry with them valuable nutrients (Deegan 1993; Varpe and Fiksen 2005; Marczak et al. 2007; Bauer and Hoye 2014). Despite its potential importance, migration‐driven ecosystem connectivity is largely overlooked in, for example, spatial planning of human activities, and ecosystem models (as pointed out by, i.e., Loreau et al. 2003; Crowder and Norse 2008; Foley et al. 2010) and empirical documentation of the local ecological effects and descriptions of the underlying mechanisms is surprisingly rare (i.e., Varpe and Fiksen 2005; Bauer and Hoye 2014).

As a consequence of the inherent seasonality of most migration events, resident predators that live along the migration routes encounter a yearly opportunity to tap into resource subsidies originating from primary and secondary producers in distant ecosystems. For instance, crocodiles feed on migrating wildebeest in East Africa (Polis et al. 1997; Dobson 2009), Grizzly bears await the arrival of salmon in North American rivers (Armstrong and Schindler 2011), Eleonora′s falcons time their breeding relative to the migration peak of passerine birds crossing the Mediterranean (Walter 1979), and the massive spawning migrations of Norwegian spring‐spawning herring and Barents Sea capelin convey primary production from the open ocean to coastal ecosystems (Røttingen 1990; Varpe et al. 2005). However, with exception of a few iconic case studies, the ecological relevance of these trophic migrant–resident interactions is often not well understood. We may recognize that a resident predator population is overlapping geographically with migration routes of potential prey, but we may fail to understand how, and the extent to which, migrants and residents affect each other. Do the migrants provide a quantitatively or qualitatively significant subsidy compared to resident prey? Is the migrant prey impacting growth or reproduction of the predator? Do the predators make deliberate decisions to switch from resident prey to these types of migrants, for instance dependent on maturation and prey size? Answering these types of questions is pivotal for further advancement in our ability to understand links and coupled dynamics between populations and ecosystems, including the climatic and anthropogenic influence on distribution, connectivity, and phenology (Bender et al. 1998) (Thurber et al. 1994; Robinson et al. 2009).

**Figure 1:**
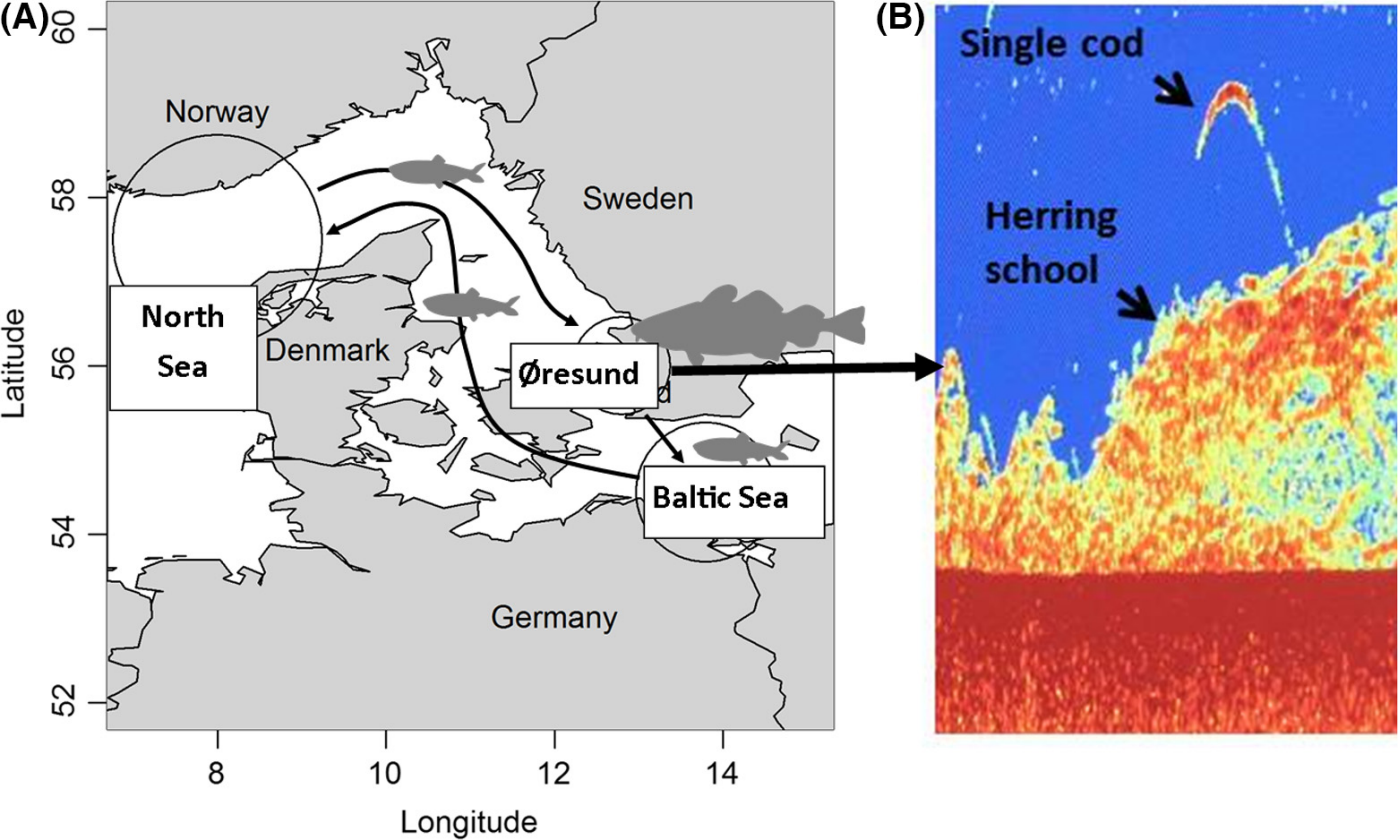
Study area (Øresund). (A) Arrows illustrate the migration routes of Western Baltic spring‐spawning herring stock between summer feeding grounds in the North Sea, Øresund where they aggregate in fall (peaking in October), and spring‐spawning area in the Baltic Sea. (B) Eco‐sounder image recorded on 2 October 2014 from image depicts a dense herring school and a single large fish (assumed to be a cod) hovering above the school.

In order to provide insight into some of these general questions, we report from a case study focusing on a local subpopulation of the Atlantic cod (*Gadus morhua*) residing in the narrow Øresund straight at the entrance to the Baltic Sea (Lindegren et al. 2013). Lindegren et al. (2010) and Svedäng et al. (2010b) indicate that the Øresund cod population is more healthy and resilient compared to surrounding cod stocks, and ascribe this mainly to the current ban on trawl fishing in Øresund. The Øresund straight is, however, not only special because of the trawl ban. Every fall about one hundred thousand tons of adult herring (*Clupea harengus*) migrate into the area and gather in extremely dense aggregations on their way to spawning grounds in the Western Baltic Sea (Fig. 1; Nielsen et al. 2001; van Deurs and Ramkær 2007; Clausen et al. 2015), potentially conveying substantial nutrient and energy influx to Øresund.

In seasonal marine environments, large emphasis has been put on the role of dietary lipids. This also applies to cod, where the size and productivity of cod stocks have been linked to lipid‐rich forage fish (Rose and O'Driscoll 2002), and liver lipid levels in late fall have been proposed as a predictor of reproductive (recruitment) success at the population level (Marshall et al. 1999). Cod store lipids in the liver and proteins in muscle tissue when food is abundant, and mobilize these resources for later reproduction or during periods of food shortage (Schwalme and Chouinard 1999). However, a recent study indicated that not only lipid quantity, but also the qualitative composition of fatty acids in the diet (i.e., the relative presence of particular types of functional fatty acids), plays an important role in reproductive performance. In particular, dietary arachidonic acid (20:4(*n*−6), ARA) has strong positive effects on the number and survival of eggs spawned in captivity (Salze et al. 2005; Røjbek et al. 2014a).

Because the fatty acid profile, laid down in the primary producers, is conserved in primary consumers and secondary consumers (Dalsgaard et al. 2003; Iverson 2009), fatty acids can also be applied as food‐web tracers, indicating the relative role of different primary and secondary producers, or as fatty acid trophic marker (FATM, Dalsgaard et al. 2003), that can trace fat from a specific prey to a specific predator. When it comes to identifying specific predator–prey interactions, problems arise if prey has indistinguishable fatty acid profiles, or if the predator feeds simultaneously on multiple prey types with distinct fatty acid profiles. On the other hand, if the FATMs of a particular prey are more or less unique and the timescale of the turnover in the lipid compartments matches the ecological timescale investigated, predator–prey links are more clear and potentially detectable (Kirsch et al. 1998; Iverson 2009).

**Figure 2:**
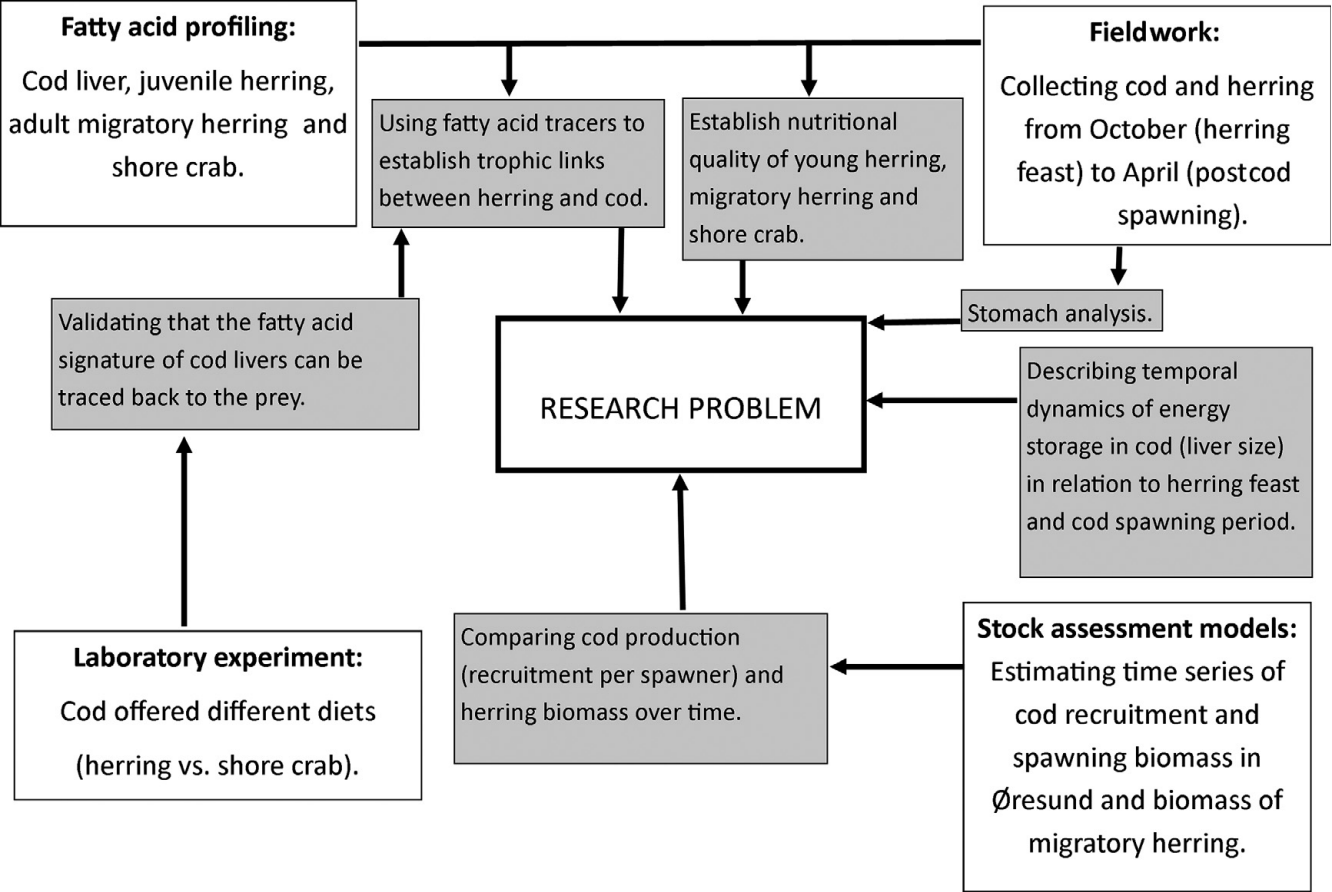
Overview of the holistic combination of techniques applied.

We here use a holistic approach that combines field investigation, laboratory experimentation, fatty acid analyses, and stock assessment models to investigate whether and how predator–prey interactions between a resident cod (the predator) and migratory herring (the prey) mediate ecosystem connectivity and influence system functioning and productivity locally (Fig. 2).

## Material and Methods

### Study area

In this study, we focus on a subpopulation of the Western Baltic cod stock residing in the Øresund straight at the entrance to the brackish Baltic Sea (Lindegren et al. 2010; Svedäng et al. 2010a,b). The spawning biomass of this subpopulation has been estimated to ca. five thousand tons (Lindegren et al. 2013) and spawning takes place from November to May (Vitale et al. 2005). Every fall about one *hundred* thousand tons of migrating adult herring (*Clupea harengus*) (>20 cm), belonging to the Western Baltic spring‐spawning herring stock (WBSS), gather in extremely dense aggregations (up to 10 million herring per NM^2^) in the northern end of Øresund, where they make a stopover on their way from feeding grounds in the northeastern North Sea to the spawning grounds near the Rügen Island in the Western Baltic Sea (Nielsen et al. 2001; van Deurs and Ramkær 2007; Clausen et al. 2015). During this autumn period, there is thus 20 times as high biomass of herring as there is of cod. For the remainder of the year, herring are found in considerably lower densities of generally smaller individual sizes. Juveniles of the WBSS herring stock do not migrate to the same extent, but disperse into the Western Baltic Sea and adjacent areas (Clausen et al. 2015; Fig. 1).

### Sampling

The field investigation was carried out between October 2014 and April 2015. Fish were trawl‐sampled in Øresund in mid‐October (when the aggregation of herring in Øresund is peaking), in mid‐November (when herring are still plentiful in Øresund), in February (peak of cod spawning period but herring have left), and in April (after cod spawning, no migrating herring). All sampling took place inside the area where herring aggregate in fall (see Nielsen et al. 2001). Hook and line were used to target larger cod, which were mostly absent in trawl hauls. Fish were kept cool, and length and liver weights were measured within ten hours of capture. A total of 20 randomly selected cod livers from fish caught in November were immediately stored at −80°C and subjected to a fatty acid (FA) analysis within three months from capture date (see the section below about FA analyses). Twenty‐two herring covering a wide size range (length stratified sampling) were selected from the October and November samples. These were also stored at −80°C and subjected to the FA analysis (see section below).

### Field investigations – energy storage dynamics and stomach content

In order to test the hypothesis that cod accumulate energy storage during the WBSS herring season in October/November and deplete storages during winter and spawning, we applied the following standardized size‐specific liver index: *LI* = [*Liver weight*]/[*Standardized fish weight*]. Standardized fish weight is calculated as 0.01 × *Length* ^2.95^, which describes the weight–length relationship fitted to all the cod in the study (wet weight in g, length in cm). The effect of fish length and season on the liver index was tested using the following ANCOVA model (alpha = 0.05): *LI* = *a*(*Length*) + *b*(*Season*) + *c*(*Length*:*Season*) + *intercept*, where *Length* is a continuous variable and *Season* a factor with four levels: October, November, February, and April. Before pooling males and females, the effect of gender was tested by including *Gender* in the model, but no significant effects were found. Stomachs were crudely analyzed in order to establish the major prey species. This involved counting whole prey items that were easily identifiable.

### Fatty acid trophic markers in cod liver and wild prey

To test the hypothesis that energy stored as fat in cod livers during the herring season originated from migratory WBSS herring, we quantified the relative contribution of 32 different fatty acids (FA) to the total lipid pool of 20 cod livers, 22 whole herring (see the section on sampling), and 8 whole shore crabs (*Carcinus maenas*). Krill constitute a key prey to herring in the northern North Sea (van Deurs and Ramkær 2007). Previous studies have reported high occurrence of the FAs 20:1(*n*−9) and 22:1(*n*−11) in Atlantic krill (*Meganyctiphanes norvegica*) and Atlantic herring, while the same FAs are rare in Baltic clupeids (Ackman and Eaton 1966; Ackman et al. 1970; Røjbek et al. 2014b). We therefore selected 20:1(*n*−9) and 22:1(*n*−11) as our fatty acid trophic markers (FATM) and predicted that the relative contribution of these to the total lipid pool would be large in migratory herring and in the livers of Øresund cod feeding extensively on these herring. WBSS herring start migrating at around the size of 20 cm and the youngest migrants tend to migrate shorter distances (Clausen et al. 2015). Intermediately sized herring are therefore expected to have less access to krill and show a relatively weaker signal of 20:1(*n*−9) and 22:1(*n*−11). A lipid pool turnover rate of *c*. 6 weeks has previously been reported for whole cod (Kirsch et al. 1998) and was found to be a suitable timescale in relation to the research questions and study design presented here. The decision to add shore crabs to the analysis was made post hoc after seeing the results of the stomach analysis mentioned in the section above. The FA analysis further provided an opportunity to investigate the nutritional quality of different sizes of herring and shore crab, that is, total lipid content and the relative contribution of arachidonic acid (ARA, 20:4(*n*–6)).

Lipids were extracted from tissue samples according to the method by Bligh and Dyer (1959). Lipid content in the extract was determined by gravimetry. The relative composition of 32 different FA methyl esters was determined from the lipid extracts. Determination was performed according to the AOCS Official Method Ce 1b‐89 with modifications using a GC‐FID (Anon 2009). The determination was performed in duplicate allowing us to evaluate the precision of the measurement. The relative contribution of each FA to the total lipid pool is given as the area% of total FA. Further details can be found in Røjbæk et al. (2012).

### Fatty acid tracers validated in feeding experiments

In order to strengthen inferences from FA analysis of wild‐caught samples, we conducted a controlled laboratory experiment and explicitly tested the hypothesis that the livers of cod foraging on adult herring from Øresund is rich in 22:1(*n*−11) and 20:1(*n*−9) compared to cod feeding solely on shore crab (or a mixture of herring and crab). At the same time, the laboratory experiment provided an opportunity to relate ARA levels in cod livers to diet composition. Sixty‐four cod (mean length = 41.5 cm, SD = 3.5 cm) were caught in gillnets in Øresund in April, when they were expected to have little fat in the liver, and separated randomly into four groups with 16 fish in each. Fish in the first group (baseline group) were sacrificed and stored at −40°C. The remaining three groups were transferred alive to three different compartments (3.5 m^3^ each) inside a large fish tank (30 psu and 10°C). Compartments were separated by net screens. Fish in the first chamber were fed three times a week with herring (mean length = 23.3 cm, SD = 2 cm) caught in early November in Øresund. Fish in the second and third chamber were fed shore crabs or a mixture of herring and shore crab, respectively (also three times a week). Both herring and shore crabs were chopped into pieces of 1 × 1 × 1 cm (±0.5 cm) before given to the cod. A total meal size of 500 g (wet weight) was served to each chamber at each feeding session. At the tenth feeding session, all three meals were enriched with vitamin B (Hansen et al. 2007). After 20 feeding sessions, all cod were sacrificed and livers removed and stored at −40°C until FA analysis. From each group of 16 cod, we randomly selected 12 individuals for the FA analysis. The FA analysis followed the same procedure as described in the section above. ANOVA with Tukey's post hoc pairwise comparison (*α* = 0.05) was applied to test for significant differences between groups (baseline, herring feed, crab feed, and mixed feed).

### Stock assessment – population dynamics

To evaluate whether the population dynamics of cod in Øresund follow trends in the WBSS herring stock, we relied on stock assessment data (Lindegren et al. 2013). Cod recruitment of age‐1 fish (*R*) and spawning stock biomass (*S*) was calculated using extended survivorship analysis (XSA, Shepherd 1999) on data (1997–2013) specifically from Øresund. Details about the stock assessment in Øresund are documented in Lindegren et al. (2013). The biomass of the adult migrating fraction of the WBSS herring stock was calculated using stock numbers (age‐2 and older) and weight‐at‐age as reported by the Herring Assessment Working Group (Anon 2015) (note that stock properties apply to January 1). Covariation of cod recruitment per ton spawner (*R*/*S*) and herring biomass (*H*) was analyzed using a simple linear regression model on the form: ln(*R*_year i_/*S*_year i‐1_) = *a *× ln(*H*_year i‐1_) + *intercept* (*α* = 0.05).

## Results

### Energy storage and stomach content of cod

**Figure 3:**
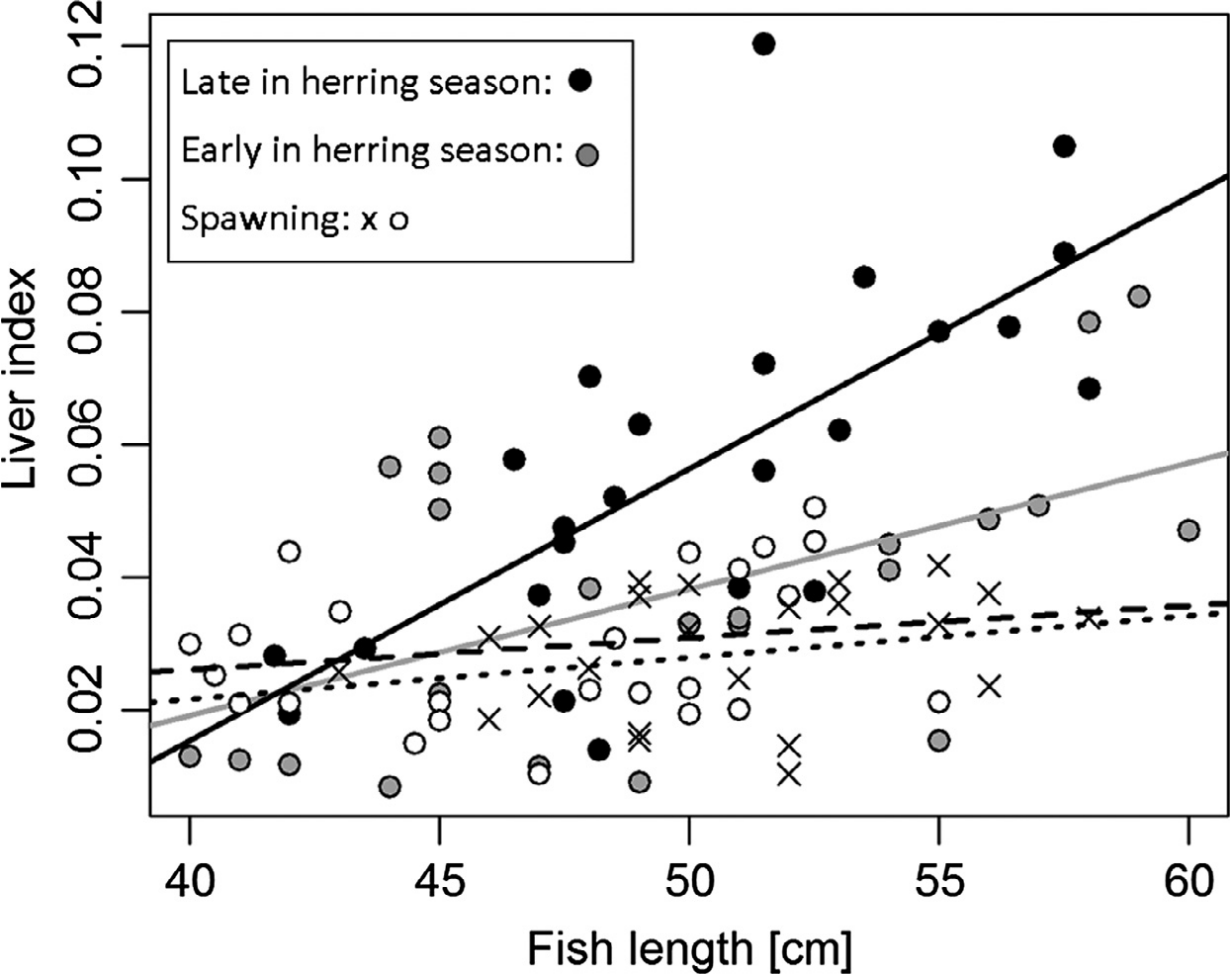
Seasonal patterns of the liver index of cod from Øresund. The liver index is used as a proxy of the size of the energy storage (liver index is equal the weight of the liver relative to the body mass). Each symbol represents an individual cod. Symbol shapes and colors correspond to sampling month. Gray dots and gray trend line: October (early in the herring season); black dots and black trend line: November (late in the herring season); white dots and broken trend line: February (peak spawning period); crosses and dotted trend line: April (postspawning).

The ANCOVA model revealed a significant interaction between fish length and season and overall positive effects of fish length (*P* < 0.001) and season (*P* < 0.001) (ANCOVA: *P* < 0.001, *r* ^2^ = 0.51, *F*_7,90_ = 15,36) on the liver index. The liver index was in general higher for fish caught in November (late in the herring season) compared to October (early in the herring season) and February/April (peak‐ and postspawning, Vitale et al. 2005) (Fig. 3). The effect of fish length on liver index was only significant for November, where large cod possessed a significantly larger liver (relative to body mass) compared to small cod. When data from November were removed from the ANCOVA model, the effect of length was no longer significant (*P* > 0.05). October was characterized by large variability in liver index between individuals. Only cod between 40 and 60 cm were included in the month‐to‐month comparison, as fish outside this size range were only represented in one of four sampling months. Small and large clupeids dominated the diet in fall (October/November) followed by shore crab, whereas shore crab dominated in winter/spring (February and April) followed by predominantly small clupeids (the frequency of empty stomachs was particularly high in February). Other prey items such as shrimps and small flatfishes were occasionally found, but even when grouped together as “other,” they were of minor importance compared to clupeids and shore crab. A maximum of 13 herring were found in a single stomach, and for shore crab, the maximum number was eight.

### Nutritional quality of migrant versus resident prey

**Figure 4:**
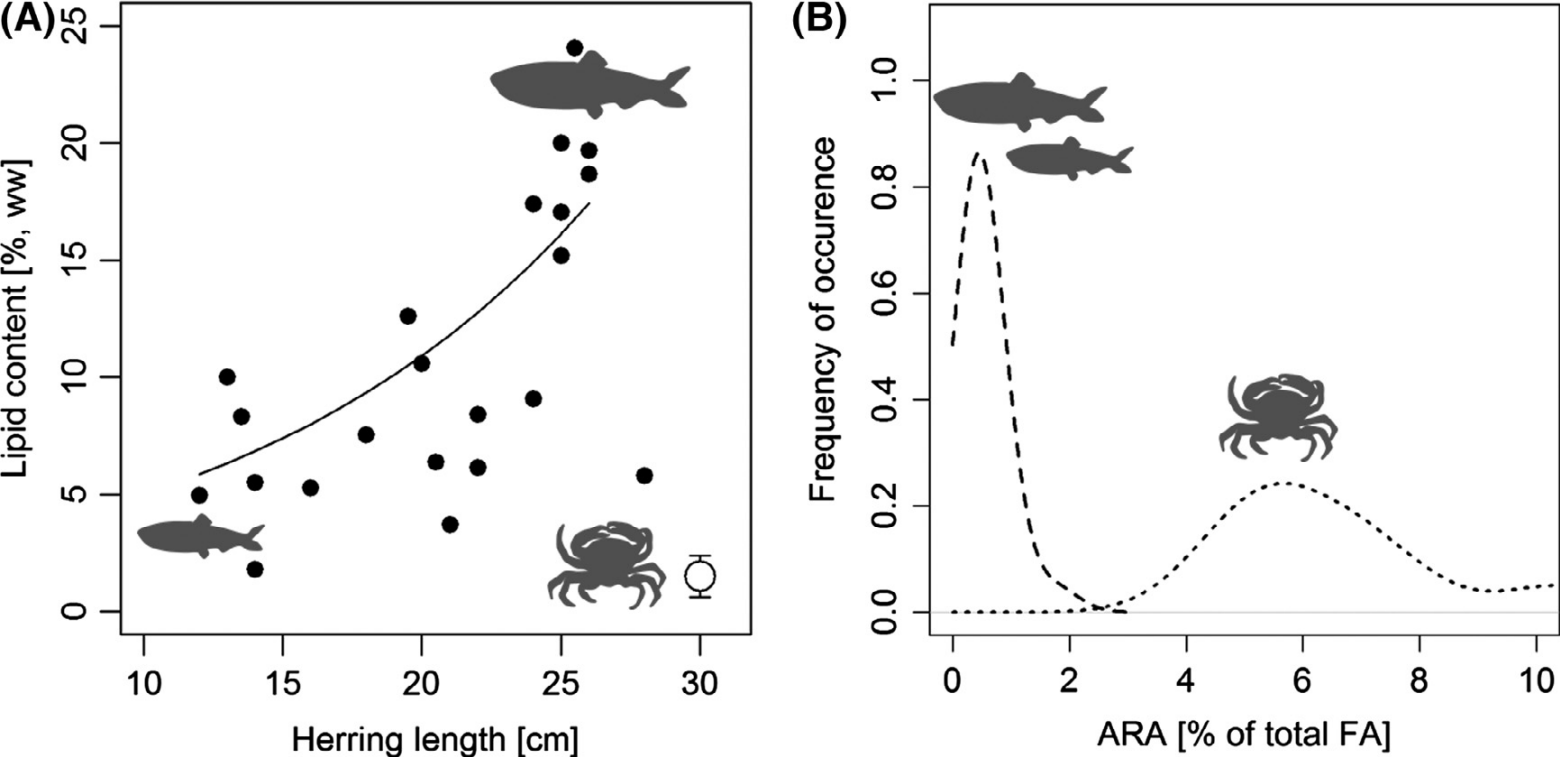
Prey quality of migrant prey (adult herring) and resident prey (juvenile herring and shore crab). (A) Lipid content (given as the % of thawed wet weight) as a function of herring size. The trend line is fitted using the following linear regression model: ln(Lipid%) = 0.08 * *Fish length *+ 0.58 (*P* = 0.003, *r* ^2^ = 0.37, *F* = 11.5, df = 20). The white symbol in the lower right corner represents the corresponding average lipid content of shore crab (whiskers represents 2 times s.e.). (B) Density plot depicting the ARA content (given as % of total FA) in herring (broken line) and shore crab (dotted line) as frequency distributions.

**Figure 5:**
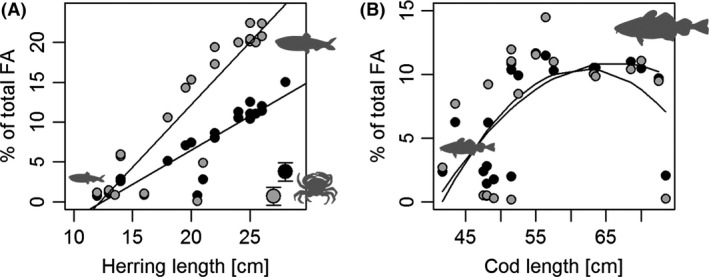
Using fatty acid trophic markers to identify trophic links. The area % of two fatty acids, 20:1(*n*−9) (black) and 22:1(*n*−11) (gray) in herring (A), shore crab (large symbol in the lower right corner of graph A) and cod (B) caught in Øresund during the herring season. For herring and cod, individual values are provided (% of the total lipid pool) and plotted against fish length. For shore crab, only the average value is presented (*n* = 8) with SD whiskers. All model fits were significant according to an alpha of 0.05.

Lipid content (% of thawed wet weight) of herring increased exponentially with the length of the herring and reached *c*. 20% in adult migratory WBSS herring (Fig. 4). In comparison, resident shore crab contained 1.5% and nonmigratory juvenile herring (<20 cm) 7–8%. The difference between crab and juvenile herring was also significant. The relative contribution of ARA to the total lipid pool (area% of total FA), on the other hand, was significantly higher in shore crab (*c*. 6.5%) compared to herring of any size (*c*. 0.5%) (Fig. 5). See Appendix 1 for an overview of all the 32 different FA methyl esters extracted from the lipid extracts.

### Tracing the fat of migratory herring in cod liver

**Figure 6:**
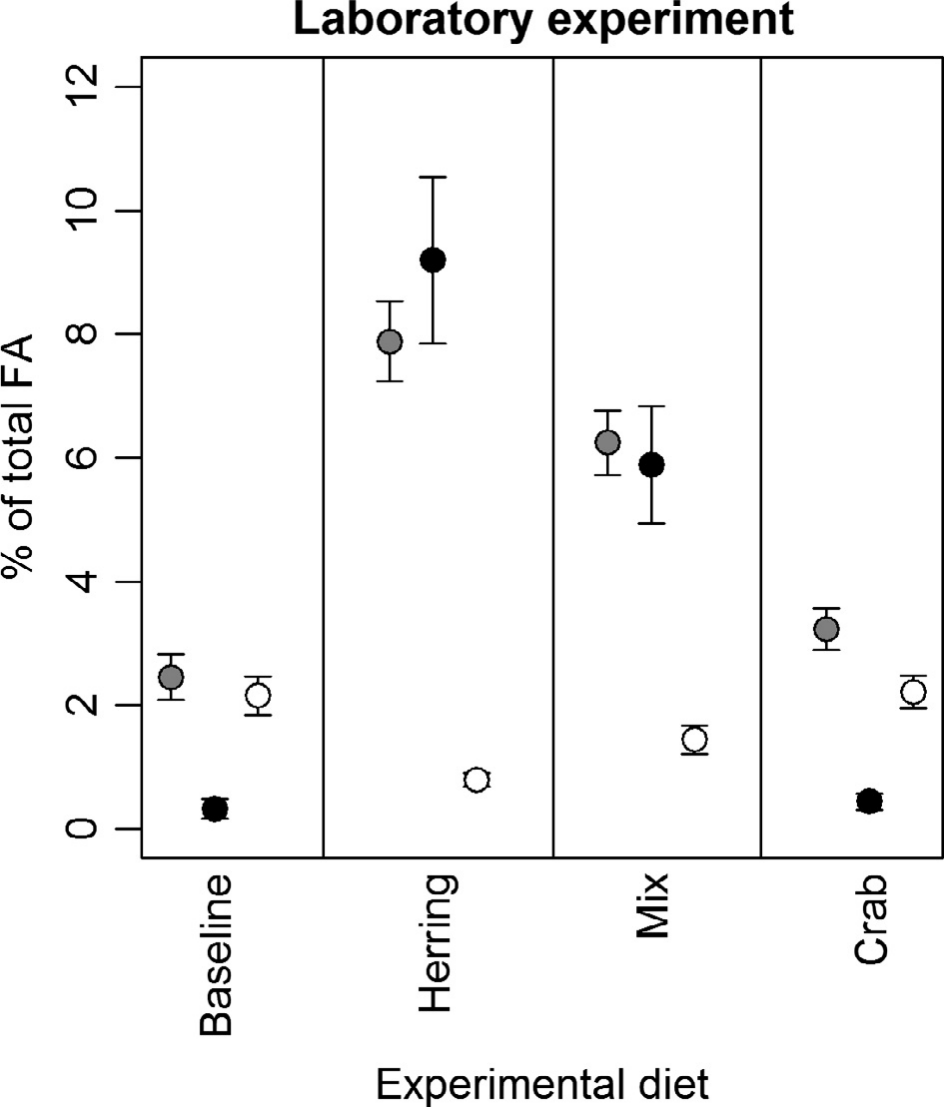
Laboratory experiment confirming the utility of fatty acid trophic markers. The figure shows the average relative contribution of 20:1(*n*−9) (gray), 22:1(*n*−11) (black), and ARA (white) to the total fatty acid pool of the cod livers from the baseline group (fish killed before the experiment) and three different experimental diets: Herring from Øresund caught during the herring season, shore crabs, and a mixture of shore crab and herring. Each symbol represents a mean value (*n* = 12), and whiskers represent 2 times s.e.

As predicted, the area% of our candidate fatty acid trophic markers (FATM) 20:1(*n*−9) and 22:1(*n*−11) were high in large herring and livers from large cod (>50 cm) caught in Øresund during the herring season (October/November). In contrast, the relative contribution of these FAs to the total lipid pool was very low in small herring and shore crabs (Fig. 5). The laboratory experiment supported the results from the field investigation by showing that livers from cod caught in April (postspawning) contained very low levels of 20:1(*n*−9) and 22:1(*n*−11) (the baseline group in the experiment), but after 6 weeks in captivity, the two groups of cod that during this period received a diet containing herring (caught in Øresund in early November) displayed a highly significant increase in 20:1(*n*−9) and 22:1(*n*−11) (Fig. 6). In summary, the baseline group was nearly identical to the crab group (ANOVA and Tukey's pairwise comparison: p_20:1(*n*–9)_ = 0.14, p_22:1(*n*–11)_ = 0.99, p_ARA_ = 0.99, *F*_3,43_ = 103,3, *F*_3,43_ = 104,6, *F*_3,43_ = 26,74), supporting the notion that shore crab represents a major component of the cod diet in Øresund during the periods when most herring is absent from the system (i.e., in April when baseline the experimental cod were caught). All other pairwise comparisons between groups were significant (p_20:1(*n*–9)_<0.001, p_22:1(*n*–11)_ <0.001, p_ARA_<0.01). We also compared the content of the candidate FATMs between the cod fed adult migratory herring in the laboratory and cod > 50 cm caught in the field and found no significant difference between these two groups (Student's *t*‐test: p_22:1(*n*–11)_ = 0.99, p_20:1(*n*–9)_ = 0.17, *t*_18.88_ = 0.0057, *t*_14.95_ = 1.47). Hence, despite the finding that 22:1(*n*−11) is proportionally more abundant than 20:1(*n*−9) in the adult herring (Fig. 5A), they are reflected in more or less equal proportions in the livers of cod fed these herring. Lastly, the notion that shore crab represents a major source of ARA was also supported by the laboratory results, showing significantly higher ARA levels in livers from cod served shore crabs compared to cod fed herring (Fig. 6). See Appendix1 for an overview of all the 32 different FA methyl esters extracted from the lipid extracts.

### Population dynamics

**Figure 7:**
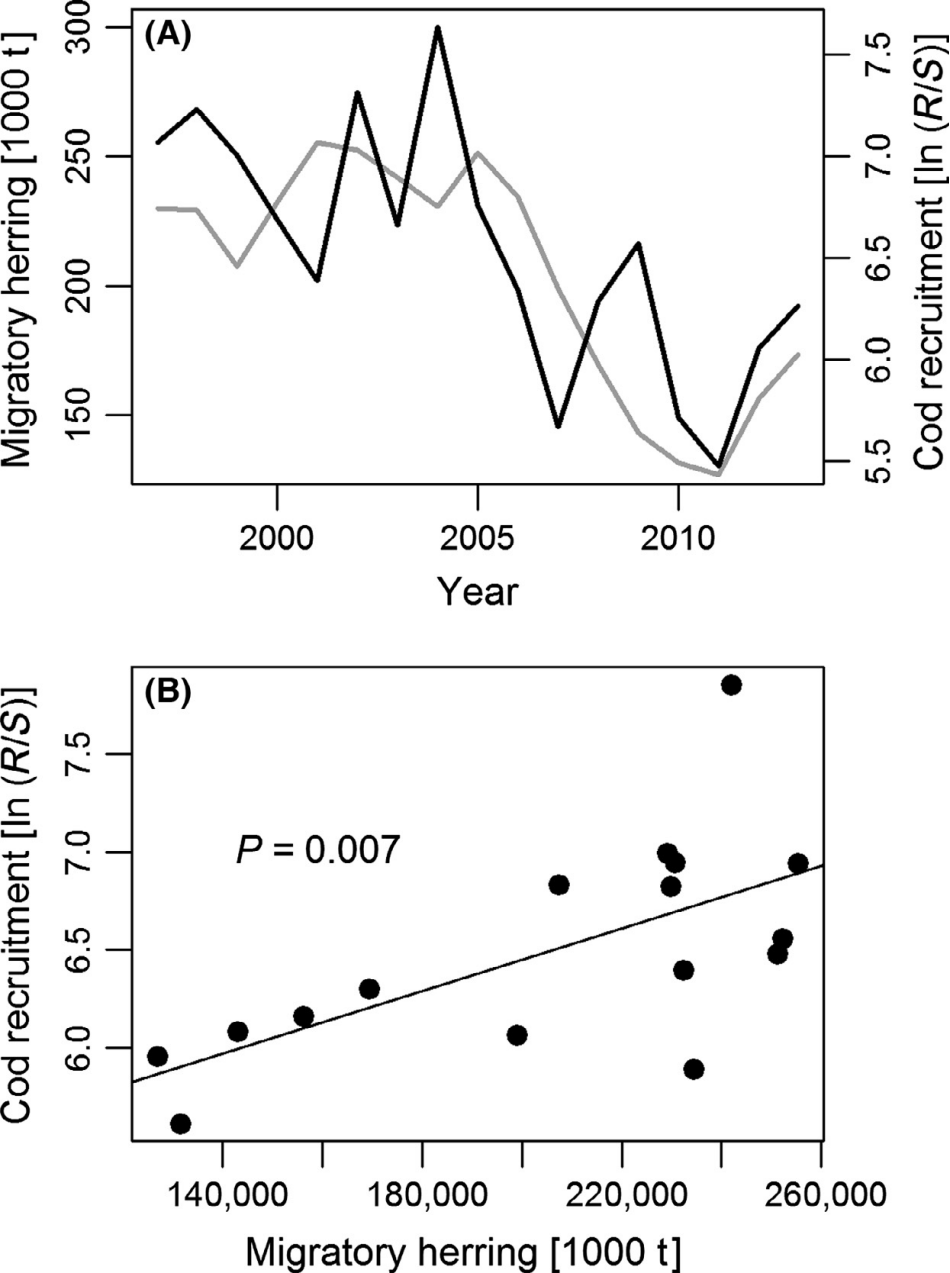
Population dynamics of cod and herring. (A) Stock dynamics of migrating adult Western Baltic spring‐spawning herring stock herring (black line), cod recruitment of age‐1 fish in Øresund (gray line), and cod spawning stock biomass in Øresund (gray line). (B) Log‐transformed cod recruitment per spawner, LN(R/S), as a function of herring biomass [tons]. The *P*‐value refers to the linear regression model (more details in method section and result section).

From the late 1990s to 2005, cod recruitment per spawner in Øresund was relatively high. In the same period, the biomass of adult WBSS herring was also large (>200 thousand tons). After 2005, the biomass of migratory WBSS herring decreased rapidly, accompanied by an equally rapid drop in cod recruitment per spawner (settling at a level only half of that found for the period from 2000 to 2005). The last two years indicate the beginning of a parallel recovery of both herring and cod (Fig. 7A). The linear stock–recruitment model showed a significant positive relationship between the biomass of migratory WBSS herring and cod recruitment per spawner (Linear regression model: *P* = 0.007, *F*_10,11_, df_1 and 14_) (Fig 7B). It is possible that cod recruitment and herring biomass covary with some external factor such as climate, zooplankton availability, or fishing quotas (i.e., Lindegren et al. 2013). However, we find this unlikely, as regulation of adult herring biomass takes place also outside Øresund and the biomass in year_i_ is the accumulated result of processes influencing between year_i‐1_ to year_i‐3_.

## Discussion

In the present study, we discovered migrant–predator interactions and substantial cross‐system lipid transportation, influencing local productivity in the transition zone between the Baltic Sea and the North Sea. We traced lipids from the herring feeding grounds in the North Sea to the cod livers in the Western Baltic Sea. Time series analysis of population dynamics indicated reproductive relevance of the herring subsidies. However, the fatty acid trophic markers (FATM) further revealed that during the herring season large cod received most of its dietary lipids from the herring, whereas smaller cod were prevented from accessing the lipid pool due to a mismatch in predator–prey size ratio. Furthermore, while the herring were extremely rich in bulk energy, they were surprisingly poor in a particular functional fatty acid.

**Figure 8:**
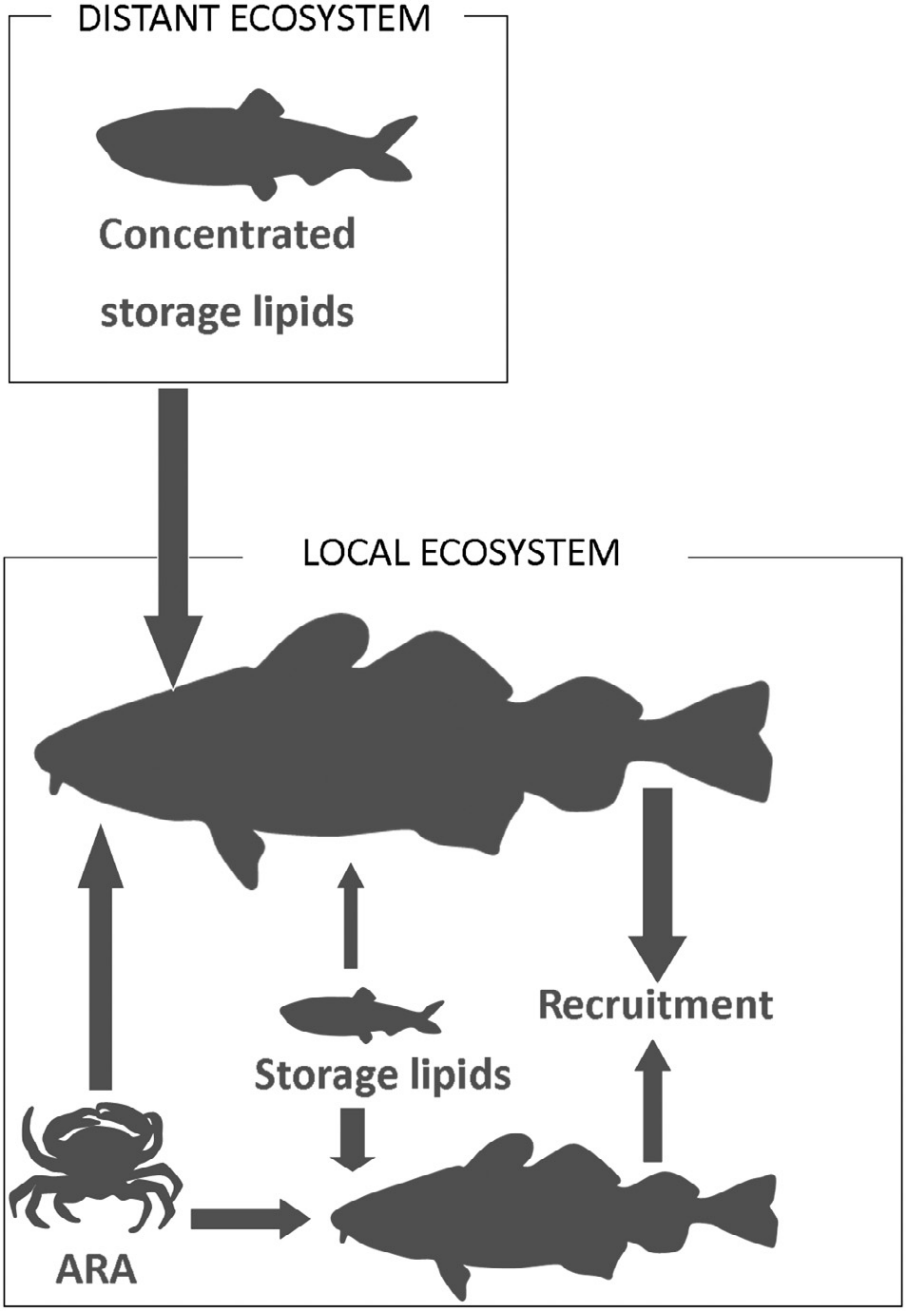
Schematic summary of the main results. Large cod (>50 cm) in Øresund receive concentrated lipids from adult migratory herring in fall. These lipids accumulate as reserves in the liver and boost reproductive output later in the season. Small cod acquire lipids from small herring that contain considerably less lipids compared to the adult migratory herring. Hence, small cod have relatively smaller lipid reserves compared to large cod. However, herring is not the primary source of the important arachidonic acid (ARA); instead, a large proportion of the lipids in shore crab (another key prey species) consists of ARA, indicating that cod need to maintain a balanced diet in the month preceding spawning, where bulk lipids and energy come from herring and ARA from shore crab.

Dietary lipids have been found to have strong effects also on cod from the Barents Sea, where cod prey on capelin when available (Bogetveit et al. 2008) and liver weight and recruitment of cod vary positively with the availability of capelin prey (Marshall et al. 1999; Hjermann et al. 2007). However, FATMs suggested here that the impact of the migrants on the predator population is potentially determined by several additional factors. For instance, the interactions were restricted to the segment of the cod population above 50 cm of length, and interestingly, we also discovered that the arachidonic acid (ARA), which plays a physiological role in cod reproduction (Røjbek et al. 2014), constituted <1% of the total lipid pool in the otherwise extremely energy‐rich migratory herring. Instead, a resident prey species, the shore crab, was found to be rich in ARA, and both stomach data and the FA composition of cod livers in the “baseline group” and “crab treatment” of the laboratory experiment indicated that this species is an important component of the diet of cod in this area. Figure 8 summarizes the main findings and forms a base for the following discussion.

**Figure 9:**
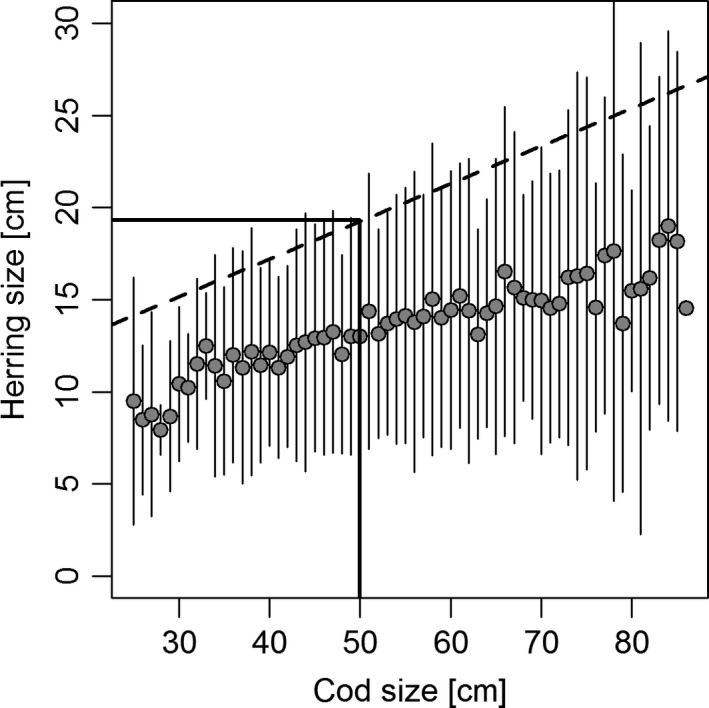
The relationship between cod size and the length of herring found in cod stomachs. Data were collected in the Baltic Sea between 1963 and 2014 and contain 3588 observations. Symbols are average prey lengths for each centimeter group (whiskers are standard deviations). The dashed regression line defines the maximum prey size and is fitted to the upper 5% confidence limit of the average values. The horizontal and vertical lines show that cod smaller than 50 cm are unlikely to feed on herring at or above 20 cm.

It is generally acknowledged that predators are restricted in the size range of prey they can consume and that this has consequences for food‐web structure and ecosystem dynamics (Warren 1996; Emmerson and Raffaelli 2004; Barnes et al. 2010). Furthermore, recent studies have suggested that even within the size range consumed by the predator, the energetic reward per capture may be considerably higher for larger prey, and shifts in average prey size may therefore affect trophic energy transfer efficiency (van Deurs et al. 2015; Golet et al. 2015). In the present study, we provide evidence that some of the same processes may determine the strength of ecosystem connectivity facilitated by trophic migrant–resident interactions. We found that the migratory herring were not only relatively large and highly abundant, but also extremely lipid rich (Hislop et al. 1991). Furthermore, the rapid increase in liver mass (fat storage) of large cod during the herring period and FATMs in cod livers pointing to consumption of migratory herring strongly suggest that large cod are preying deliberately on migratory herring, whereas smaller cod are restricted to smaller nonmigratory prey. An extensive dataset on cod stomach content from the Western Baltic Sea strongly supports that cod are highly unlikely to consume herring larger than 1/3 of their own length (Fig. 9). The fact that predator–prey size ratio defines the strength of the migrant–resident interaction emphasizes the importance of taking into account population size structure in attempts to understand and predict the strength of cross‐system fluxes of energy.

Although addressed sporadically in the literature since the 1970s, the potential ecological importance of nutritional quality and balanced diets has only recently been broadly recognized among ecologists, and it is still a largely unexplored research field (Pulliam 1975; Cruz‐Rivera and Hay 2000; Simpson et al. 2004; Jensen et al. 2012). For example, general nutritional theory states that some nutritional elements can be synthesized by the organism, whereas other nutrients are essential in the sense that they are required in the diet to achieve survival, optimal growth, or reproduction (Brett and Müller‐Navarra 1997). Balancing the uptake of dietary proteins and lipids is also essential in order to fulfill the life cycle tasks on which natural selection acts. For instance, the optimal physiological state depends on whether the individual animal is preparing for overwintering, gonad development, or somatic growth (Jørgensen and Fiksen 2006). An individual preparing gonad development should benefit more from a lower protein:lipid ratio than an individual prioritizing growth. However, lipids also serve to increase protein conversion efficiency, because energy can be drawn from lipids while proteins are retained as building blocks, meaning that even if a fish allocates all its resources to growth during certain periods of life, it should not completely avoid lipids in its diet (Silverstein et al. 1999; Tocher 2003).

While the migratory herring were rich in bulk lipids, ARA levels were low compared to shore crab. ARA is a precursor for eicosanoids, such as prostaglandins (that play a role in the late phase of gonad maturation and ovulation) and the amount of ARA in the diet has a strong positive effect on the number and survival of eggs spawned by cod in captivity. Increasing dietary ARA for female cod from 1 mg/g ww to 4 mg/g resulted in a doubling of the number of eggs produced by cod in captivity (Røjbek et al. 2014). However, the link between lipids and the reproductive cycle of cod is a complex one, potentially involving several different functional FAs (Røjbek et al. 2012). For example, eicosapentaenoic acid (20:5(*n*−3), EPA) is also a precursor for eicosanoids and therefore compete for the same membrane receptors. However, it appears so that eicosanoids produced from EPA is less active than those produced from ARA. Hence, also the ratio between EPA and ARA may play a role in the final stages of the reproductive cycle (Bell et al. 1986).

Growth studies have shown that a diet containing a protein–lipid ratio of *c*. 2.5 is optimal for growing cod (Grisdale‐Helland et al. 2008). Assuming a protein content of adult herring of 20% (Lawson et al. 1998), the protein–lipid ratio is approximately 1, considerably below the growth optimum. However, as the herring aggregate in Øresund between the somatic growth period and the gonadal growth period of the cod, the timing of the herring consumption may fulfill a need for large lipid storages prior to the peak spawning period in February (Vitale et al. 2005), rather than fulfilling the requirement for optimal growth. Had the timing been different, for instance spring time when somatic growth is a first priority, then a protein–lipid ratio of 1 in the herring may have been less of an advantage.

The WBSS herring stock mixes with North Sea herring at the feeding grounds, and extensive research effort has made it possible to separate the two stocks (i.e., Clausen et al. 2015) and provide stock‐specific management advice to ensure diversity of herring populations in the area, each of which may sustain local predator populations and small‐scale fisheries (Waldo et al. 2013). The voluntary minimum landing size in the Øresund cod fishery is 45 cm (the general minimum size in the Baltic Sea is 38 cm), fishing mortality of Øresund cod is relatively low, and bottom trawling is prohibited (Anon 1932; Lindegren et al. 2010, 2013). We therefore argue that a combination of the cross‐system flux of herring subsidies each fall, and the prevailing management regime (of both WBSS herring and cod in Øresund) underpins the productivity of the Øresund cod population, while neighboring cod populations are under pressure (Lindegren et al. 2010; Svedäng et al. 2010b). Under a different management scenario, however, cod >50 cm may have been fewer in Øresund (i.e., Sinclair et al. 2002) or the WBSS herring stock smaller, and as a consequence, the migrant–resident interaction may have been reduced and reproductive performance degraded, as less lipids would have found their way to the cod population.

It has previously been suggested that prey subsidies, by movement of either prey or predators, often enhance predator production beyond what local resources can support (i.e., Polis et al. 1997; Bauer and Hoye 2014). The present study supports this notion and thereby highlights the importance of addressing migrant–resident interaction in ecosystem models, conservation initiatives, infrastructure planning, and fisheries management. Additionally, the present study discovered that the magnitude and ecological profits of cross‐system fluxes of subsidies in migrant–resident systems are potentially constrained by the size structure of the resident predator population and the nutritional quality (as opposed to a narrow focus on caloric quantity) of the migrants. Ultimately, this study should draw our attention toward the overarching question: What are the consequences of the disappearance and reduction in migrations worldwide?

## Conflict of Interest

None declared.

## Supporting information


**Appendix 1**. Average values for all the 32 different FA methyl esters extracted from the lipid extracts.
